# The development of a digitising service centre for natural history collections

**DOI:** 10.3897/zookeys.209.3119

**Published:** 2012-07-20

**Authors:** Riitta Tegelberg, Jaana Haapala, Tero Mononen, Mika Pajari, Hannu Saarenmaa

**Affiliations:** 1 Digitarium: The Digitisation Centre of the Finnish Museum of Natural History and the University of Eastern Finland, School of Computing, SIB-labs, Joensuu Science Park, Länsikatu 15 (P.O. Box 111), FIN-80101 Joensuu

**Keywords:** Digitisation, imaging, natural history collections, service packages, out-sourcing, mass-digitisation, automation, logistics, costs, IPR

## Abstract

Digitarium is a joint initiative of the Finnish Museum of Natural History and the University of Eastern Finland. It was established in 2010 as a dedicated shop for the large-scale digitisation of natural history collections. Digitarium offers service packages based on the digitisation process, including tagging, imaging, data entry, georeferencing, filtering, and validation. During the process, all specimens are imaged, and distance workers take care of the data entry from the images. The customer receives the data in Darwin Core Archive format, as well as images of the specimens and their labels. Digitarium also offers the option of publishing images through Morphbank, sharing data through GBIF, and archiving data for long-term storage. Service packages can also be designed on demand to respond to the specific needs of the customer. The paper also discusses logistics, costs, and intellectual property rights (IPR) issues related to the work that Digitarium undertakes.

## Introduction

In Finland, the 6 largest public natural history museums contain an estimated 22 million specimens, of which 12% have been digitally catalogued (i.e., minimally digitised). In addition, private collections contain up to 8 million specimens. It has been estimated (Pelkonen et al. 2009) that unless digitisation productivity is dramatically increased, it will take about 1,000 person years of effort to digitise these collections. Thus, in 2010, Digitarium, the Digitisation Centre of the Finnish Museum of Natural History and the University of Eastern Finland, was established in Joensuu, Finland. Digitarium aims to speed up the digitisation process through an efficient production line and knowledge management of expertise on digitisation. The main idea is to selectively outsource mass digitisation from major museums into a dedicated service centre that works in close cooperation with museum customers. In most cases this also includes return transportation of the material to the service centre.

Special features of the production process at Digitarium are imaging of all material, XML-based data management, and a distributed workflow that can employ distance workers. Automation of imaging will produce large quantities of material ready for data entry. In addition to offering the employees working on data entry the option of working from home or from a library, remote access also provides an opportunity for crowd sourcing (Howe 2006, Flemons 2011, Flemons and Berents 2012). Crowd sourcing also functions as a means of promoting free and open access to national collections.

This document briefly outlines the process of digitisation as it is being implemented at Digitarium. In addition, the paper describes the approach used to develop service packages for customers, which are formed by connecting the steps of the digitisation process in a way that the customer requires. Finally this paper visits the issues of logistics, costs, and intellectual property rights (IPR), which are important in an outsourced operation.

## Process steps

**Figure 1.**
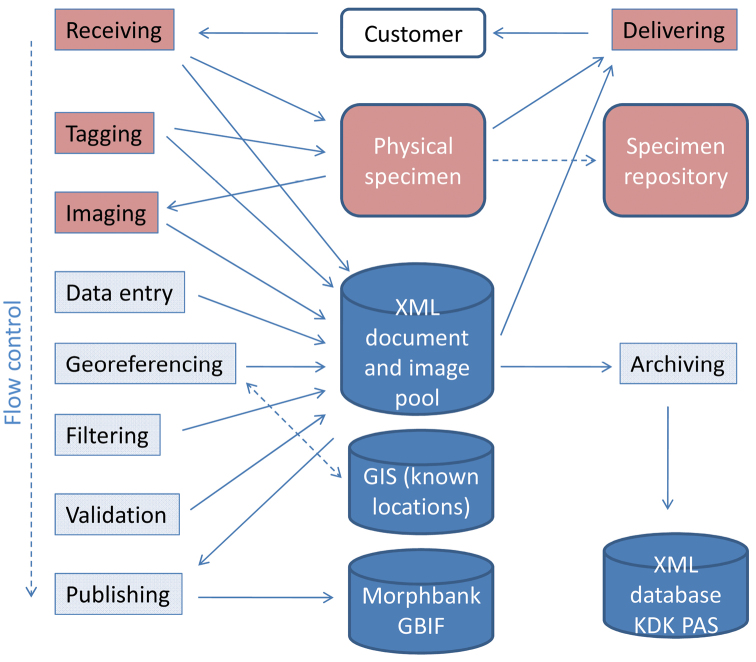
Functional model of the digitisation process. The steps pictured in red require handling of the physical specimens.

The steps of the digitisation workflow process are illustrated in the functional model shown in Fig 1. The steps from Receiving to Imaging require some handling of the physical samples, whereas steps from Data Entry onwards can be distributed through the internet to the best available agent. All steps of the workflow process can be executed asynchronously, although their logical order is somewhat fixed. The process is described in more detail in Lehtonen et al. (2011).

**Figure 2.**
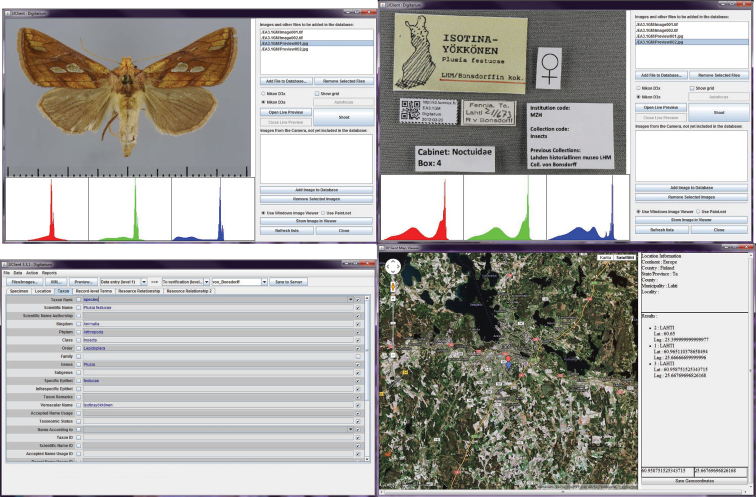
Selected windows of the digitisation workbench.

The process and workflow described below is driven by a dedicated software workbench (Fig. 2). This tool has been written by Digitarium in Java, and it runs on Windows. The workbench manages all data in the form of XML documents, and drives the digital cameras for imaging. It can also be used for distance work, and through SSH it can remotely retrieve and write the XML documents pertinent to each step in the workflow. The produced XML data conforms by the Darwin Core and Dublin Core standards.

The metadata describing datasets (i.e., groups of Darwin Core XML documents, as well as orders by customers) are stored in XML files using the Ecological Metadata Language EML (Fegraus et al. 2005), which is a standard for describing datasets in the biodiversity science community.

### Receiving

Digitarium does not manage collections of its own, but is a shop for digitising materials for “customers” — that is, from museums and other institutions located elsewhere. The customer institute selects material for digitisation based on their own prioritisation. The received material and the procedures used for digitising are described in EML.

### Tagging

**Figure 3.**
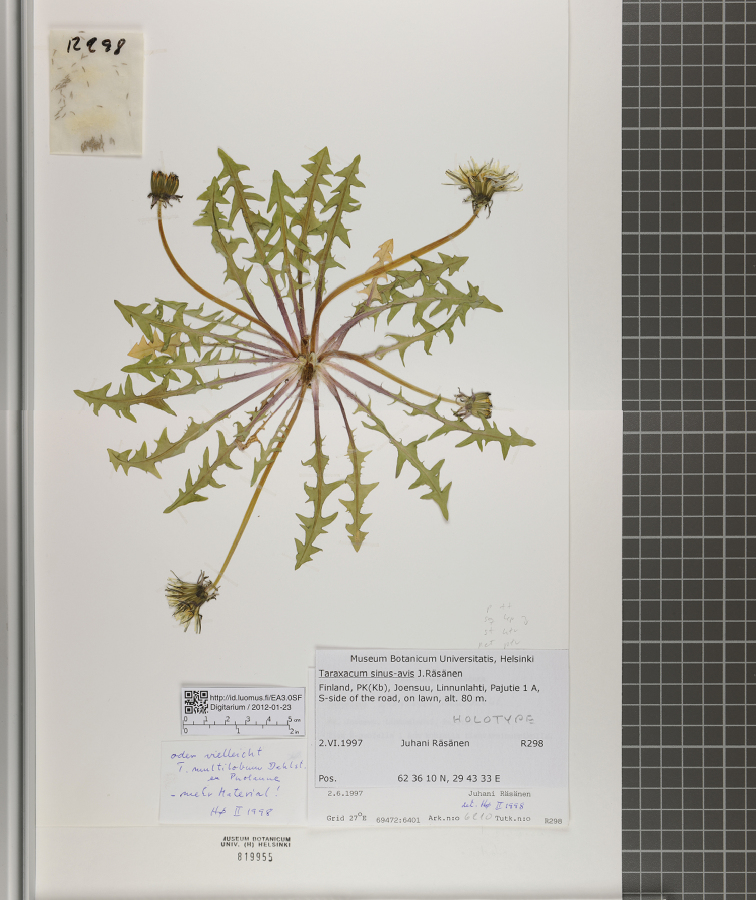
Image of a plant sheet stitched together from two parts – their boundary is barely noticeable in the middle of this sample. Notice the two-dimensional barcode, and a resolvable unique URI of the specimen details.

Each sample is tagged with a label containing globally unique identifier in the form of an HTTP URI and a two-dimensional barcode. See Fig. 3 for an example. The URI can be resolvable if it is made to point to the collection database management system of the customer.

### Imaging

The two main types of specimens that are digitised at Digitarium are plant specimens and insect specimens. A plant sheet is imaged in two pieces (see Fig. 3) with a high-end digital camera (i.e., a Nikon D3x, 24 megapixels). This way, a relatively high-quality resolution of 450 dpi over the entire sheet can be achieved at a relatively low cost. The two pieces are later joined using a panorama image stitching application based on our own algorithm, which is tuned for this kind of images. In the case of insect samples, the specimen and the labels are imaged separately with a 12 megapixel camera (i.e., a Nikon D3s using a Nikon AF-S MICRO NIKKOR 105 mm 1:2.8 objective and extension rings for the smallest objects). As the cameras are calibrated daily, no colour swatch has been included in the images. Our digitisation workbench drives all steps of image capture and annotation and all details of the imaging event and results are automatically stored in an XML document.

### Delivery and optional specimen repository

After successful imaging, the specimens are returned to their institutions. Specimens can also be stored at Digitarium’s repository for either short or long periods of time. This is an option for collections that are not under active study, and for excess specimens.

### Data entry

The data from the specimen labels is entered manually from the images using our digitisation workbench and the vocabulary of the Darwin Core data exchange standard. In this step, we need to separate the “true and honest” reproduction of what has been written on the labels with the subsequent interpretation of that information. Any misspellings, abbreviations, etc., are written in the “verbatim” fields of the latest Darwin Core standard vocabulary to preserve the original data. This has been somewhat problematic, as Darwin Core has not had verbatim fields available for all possible label data such as collector name, taxonomic identification, record number, label colours, etc. Thus, separating reproduction from interpretation is not yet fully supported. This led us to propose a new term, “verbatimLabel,” for Darwin Core, although we have not yet implemented this new feature.

### Georeferencing

Geographic coordinates are often not available from specimen labels. Our software workbench contains a function to retrieve them automatically using the web services of GeoLocate (2005). We only use the estimated latitude, longitude, and coordinate uncertainty in meters for point localities. When grid coordinates from the old Finnish national system (called “YKJ”) are available, they are automatically converted into WGS-84 geographic coordinates using the point-radius method; this conversion can be well documented in Darwin Core.

Georeferencing is an optional step. It can be done by Digitarium simultaneously with data entry or verification, but it can also be left for the customer or remote expert to complete, if so agreed.

### Filtering

Before publishing the data and images, the filtering of certain details such as coordinates of localities of endangered species may be necessary. For textual and numeric data, this can be done automatically based on the entered species names stored in the metadata of the dataset. Two versions of the XML file are retained: filtered and unfiltered. These details need to be masked manually from the image. Optionally, the customer may want to perform this step.

### Validation

A final check of the data entry, georeferencing, and filtering is made by an experienced staff member. However, as the customer often wants to validate the digitisation result, all validation can be left to the customer.

### Delivery of data

The data is delivered to the customer in the Darwin Core Archive (DwC-A) format (Wieczorek et al. 2012), which has been endorsed by Biodiversity Informatics Standards (TDWG) (2009). Other delivery formats are available depending on the requirements of the customer. Furthermore, as the customer usually wants to have checkpoints for the work, intermediate data deliveries are often made. Delivery of the digital images has not yet taken place, as so far the customers have preferred Digitarium to host them.

### Publishing

The collection data from the latest XML document version, as well as the images, are imported to Digitarium’s Morphbank database service and Digitarium’s GBIF IPT service. From there they are published, as agreed with the customer; if publication has not been agreed upon, the data and images remain private, and are available only to the customer and for Digitarium’s internal use.

The Morphbank service, a part of the global and Nordic collaboration, is available at http://morphbank.digitarium.fi/. Morphbank is an image database tool designed particularly for natural history specimens and annotations made to them (Morphbank 2011). Morphbank provides permanent publication: after the preset publishing date has passed, the objects cannot, even in principle, be removed from the service. All Morphbank objects have stable short URIs that can be reused elsewhere.

The GBIF (2011) Integrated Publishing Toolkit (IPT) is a service for hosting biodiversity data that is intended to be shared globally. Its purposes at Digitarium are to produce the EML and DwC-A for all the datasets, and when agreed with the customer, to publish collection and specimen-level data thus promoting Digitarium’s services. The IPT hosting service has also been required by several smaller museums and collections that do not have the infrastructure to connect with GBIF directly.

### Archiving

All the XML documents and images will be retained indefinitely, first on Digitarium’s Metacat (NCEAS 2012) service and eventually with the long-term archival service of the National Digital Library (2010). These archive functions are still under development.

## Packaging of the services

The services described here are designed in cooperation with the customers to be flexible and meet the unique requirements of different clients.

Prior to each digitisation job, a formal agreement is made in terms of the details of the digitisation process, costs, and time frames. When negotiating the agreement, customers are informed of the option of customising the digitising services at Digitarium. In the most basic case, the workflow will include steps from Receiving to Imaging. Data Entry will only include the actual information from the labels attached to the specimen, and basic interpretation that aids in later data discovery such as taxonomic group and country. Georeferencing, data filtering, and publishing may be left out, as the customer may want to perform these steps. However, the quality of the images and the technical correctness of the data entry will be verified.

In a more complete service package, descriptive data entry with full interpretation of taxonomic and locality details, georeferencing, and verification of the data will be included. Misspellings and unclear text will be retained in the “verbatim” field of Darwin Core, though. Dates and timings will be written following the ISO 8601:2004(E) standard. Country codes, institutional codes, and collection codes will be included.

In an “all-included” service package, all the steps shown in Fig. 1, as well as additional filtering, publishing, and archiving services, are included in the service. This is the most suitable method for the digitisation of an entire collection. The customer still has the opportunity to follow the process, sign off on the quality of products, and give scientific guidance. Entirely customised service packages can also be designed when needed so that resources and funding can be used to most directly answer the needs of a particular customer.

It is expected that, in the future, customers would want to monitor the progress of their digitisation jobs. For this purpose, a tracking and metadata system for the planning and scheduling of digitisation work is being prepared.

Customers are also able to participate in data entry first hand. In order to facilitate such collaboration, training on the Digitarium process can be included in the service. The aim is to produce repeatable and quality data, regardless of where the actual data entry takes place.

Finally, if a customer wants to operate these services entirely in-house, Digitarium can offer a turn-key package that includes the equipment needed to run the imaging and data entry processes. In this way, the customer may process the most delicate specimen samples in the safety of their own institution, while following the standards brought into use at Digitarium.

## Logistics, costs, and intellectual property rights

Because the Digitarium service centre is located away from where the collections are housed, a few special issues must be taken into consideration. Quite rightly, transportation of the materials to the service centre is of major concern for the custodians of the collections. Not all materials can be considered for transportation (such as those stored in liquids). Materials that can be considered for transport must be carefully packed to ensure that they cannot move during transport. For botanical sheets this can be achieved, but requires some work. Insect collections are easier to package and transport; perhaps that is why most demands for Digitarium services have come from entomology collections. In a typical case, Digitarium retrieves an endowed entomological collection, dismantles and processes it, and then delivers it to the customer institution, neatly re-packed and ordered in small units.

Receiving material also requires the extermination of possible pests that could be damaging the collection. Therefore, upon arrival, all received material is deep-frozen in a room that is in a separate building.

Processing of the material at the service centre does not necessarily take a long time, which reduces inconvenience for the customer in terms of being separated from their collection. Tagging and Imaging can in principle be done quickly, while the data entry steps can proceed at a more flexible rate (cf. Fig 1). Overall, a two- to four-week turnaround time is conceivable based on the experience of digitisation centres operating in the cultural history domain. The volume that can be processed in such time is quite variable, though, depending on type of material, and how much automation is possible.

Moving of material between organisations also requires agreement on intellectual property rights. The agreement that is made of each digitisation job transfers the copyright to the customer when the customer has accepted the final delivery. Digitarium retains a parallel right to use the content within its own internal operations, but not for delivery to other parties. This way, it can be ensured that no duplicate copies of the same data start circulating in global portals. In the case of images hosted by Digitarium on behalf of the customer, a rather restrictive variety of the Creative Commons license, BY-ND, is currently being applied.

For the costs of the services, only preliminary figures are available, as the process is still being formed and tested in many areas. So far, about 40,000 images have been made and 10,000 samples have been fully processed. Two-thirds of these samples have been entomological, and one-third botanical. On average, a staff member has been able to produce about 40 images or data entries per day. The cost of digitisation is currently 3.99 € per image and 5.61 € for data entry of a specimen, which makes a total of 9.60 € for a fully processed sample. These costs do not include development, administration, equipment, housing, etc. We expect the costs to reduce rapidly as the process becomes increasingly streamlined and automated.

**Figure 4.**
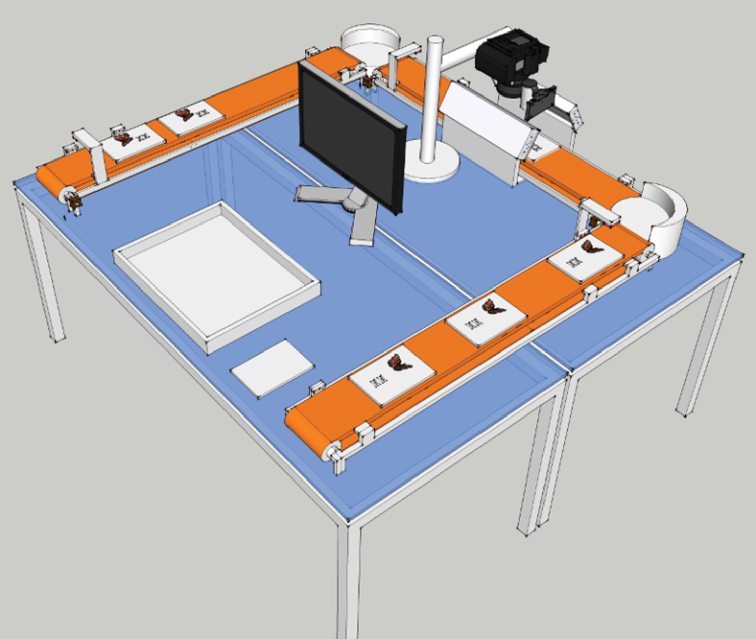
Conceptual design for automated imaging of entomological collections, which is currently being implemented at Digitarium.

What has been described above is still essentially a manual process. However, the separation of the different steps of the workflow offers a strong possibility for automation. In fact, Digitarium is in the process of building a conveyor belt system that moves the samples for automatic imaging (Fig. 4). We expect the costs of imaging to dramatically decrease when this system is in operation.

## Conclusion

Digitarium aims to accelerate the digitisation of natural history collections, both in Finland and around the world. In order to achieve industrial-scale efficiency, we are considering the aspects of quality control, economies of scale, automation of processes, cost of labour, community resources, and workflow (cf. Speers 2009).

Progress in all these areas is being made, but a full solution has not yet been delivered. In particular, the automation of imaging and related logistics is still being crafted. The fact that all material is being imaged makes it possible to distribute data entry and subsequent steps in the process to off-site workers and to rely on crowd-sourcing for Data Entry. In these ways, processing costs can be reduced and access to remote experts can be gained for purposes such as handwriting recognition, languages, and species identification. On the other hand, digitisation technicians at Digitarium are trained to produce repeatable and qualified data from all sorts of collection material.

By offering the service packages described here, Digitarium can ensure that the wishes and needs of its customers can be met. Quality assurance not only covers the images and data, but also extends to our descriptions of the process and products. In this way, customers may choose the extent of the processing they require for a particular specimen or collection based on their own prioritisation.

The Digitarium service centre is located in Joensuu, a peripheral area of Europe, where dedicated funding sources such as the European Social Fund and the European Regional Development Fund have been available to boost the economy and build infrastructure. These funding sources are available particularly to new member countries of the EU, and offer a good opportunity for building research infrastructures such as digitisation services.

We believe that the outsourcing of digitisation to dedicated service centres with decentralised processes and well-defined service packages designed in cooperation with customers can speed the digitisation process up from the current manual practices to industrial-level efficiency (GBIF 2008, Speers 2009, Berendsohn et al. 2010).
